# Supplementary material for: How Large Was the Mortality Increase Directly and Indirectly Caused by the COVID-19 Epidemic? An Analysis on All-Causes Mortality Data in Italy
Source: Int J Environ Res Public Health. 2020 May 15;17(10):3452. doi: 10.3390/ijerph17103452 (PMC7277828; doi:10.3390/ijerph17103452)
Supplement: Supplementary file 1 [file ijerph-17-03452-s001.pdf]

International Journal of
Environmental Research and Public Health

Supplementary Material

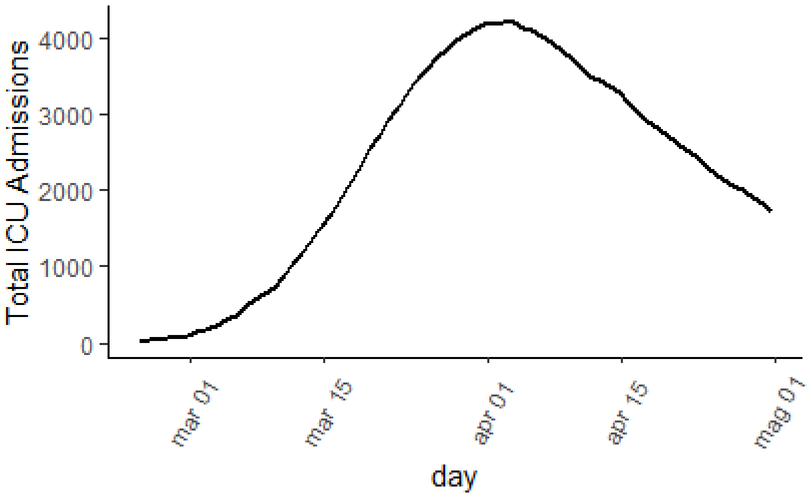

Figure S1. Time series of Total COVID-19 ICU admissions.

© 2020 by the authors. Submitted for possible open access publication under the terms and conditions of the Creative Commons Attribution (CC BY) license (http://creativecommons.org/licenses/by/4.0/).
